# Impact of Tea Processing on Tryptophan, Melatonin, Phenolic and Flavonoid Contents in Mulberry (*Morus alba* L.) Leaves: Quantitative Analysis by LC-MS/MS

**DOI:** 10.3390/molecules27154979

**Published:** 2022-08-05

**Authors:** Panyada Panyatip, Tanit Padumanonda, Chawalit Yongram, Tiantip Kasikorn, Bunleu Sungthong, Ploenthip Puthongking

**Affiliations:** 1 Department of Pharmacognosy, Faculty of Pharmacy, Srinakharinwirot University, Nakhon Nayok 26120, Thailand; 2 Melatonin Research Group, Faculty of Pharmaceutical Sciences, Khon Kaen University, Khon Kaen 40002, Thailand; 3 Division of Pharmacognosy and Toxicology, Faculty of Pharmaceutical Sciences, Khon Kaen University, Khon Kaen 40002, Thailand; 4 Division of Cannabis Health Science, College of Allied Health Sciences, Suansunandha Rajabhat University, Samut Songkhram 75000, Thailand; 5 Thai Traditional Pharmacy Program, Faculty of Pharmaceutical Sciences, Khon Kaen University, Khon Kaen 40002, Thailand; 6 Integrative Pharmaceuticals and Innovation of Pharmaceutical Technology Research Unit, Faculty of Pharmacy, Mahasarakham University, Maha Sarakham 44150, Thailand; 7 Division of Pharmaceutical Chemistry, Faculty of Pharmaceutical Sciences, Khon Kaen University, Khon Kaen 40002, Thailand

**Keywords:** mulberry leaf, tea processing, tryptophan, melatonin, phenolics, flavonoids

## Abstract

Mulberry (*Morus alba* L.) leaves from two cultivars, Yai-Burirum (YB) and Khunphai (KP), were prepared into green tea (GT) and black tea (BT). Compared to fresh leaf (FL) extract, GT and BT extracts were evaluated for their total phenolic and total flavonoid contents. Total phenolic content (TPCs) in all samples ranged between 129.93 and 390.89 mg GAE/g extract. The processing of tea decreased the levels of TPC when compared to FL extracts in both cultivars. The total flavonoid content (TFCs) in all samples was found in the range of 10.15–39.09 mg QE/g extract and TFCs in GT and BT extracts were higher than FL extracts. The change in tryptophan, melatonin, phenolic and flavonoid contents was investigated by liquid chromatography–mass spectroscopy (LC-MS). The results exhibited that tryptophan contents in all samples were detected in the range 29.54–673.72 µg/g extract. Both GT and BT extracts increased tryptophan content compared to FL extracts. BT extracts presented the highest amounts of tryptophan among others in both cultivars. Phenolic compounds were found in mulberry leaf extracts, including gallic acid, caffeic acid, gentisic acid, protocatechuic acid and chlorogenic acid. Chlorogenic acid presented the highest amount in all samples. Almost all phenolic acids were increased in the processed tea extracts except chlorogenic acid. Rutin was the only flavonoid that was detected in all extracts in the range 109.48–1009.75 mg/g extract. The change in phenolic and flavonoid compounds during tea processing resulted in the change in antioxidant capacities of the GT and BT extracts. All extracts presented acetylcholinesterase enzyme (AChE) inhibitory activity with IC_50_ in the range 146.53–165.24 µg/mL. The processing of tea slightly increased the AChE inhibitory effect of GT and BT extracts. In conclusion, processed tea from mulberry leaves could serve as a new alternative functional food for health-concerned consumers because it could be a promising source of tryptophan, phenolics and flavonoids. Moreover, the tea extracts also had antioxidative and anti-AChE activities.

## 1. Introduction

**Figure 1:**
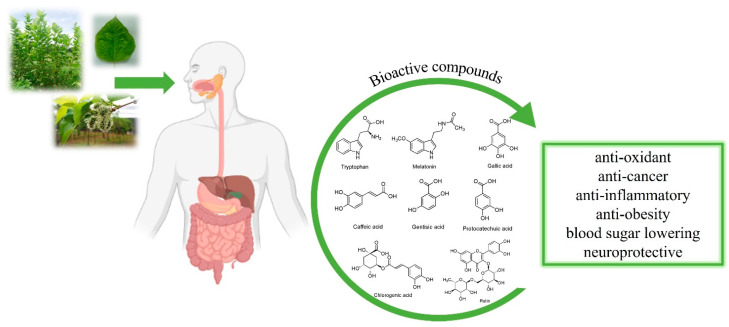
An overview of health benefits of mulberry consumption.

Mulberry (*Morus alba* L.) is a dominant species among the others that belong to the ***Morus*** genus of ***Moraceae*** family [1]. Useful bioactive constituents are found in many parts of this plant, for instance, the leaves, bark, roots, twigs and fruits [2,3]. The bioactive compounds that were found in mulberry include polysaccharides, phenolics, flavonoids, anthocyanins, alkaloids, etc. [3,4]. According to these bioactive compounds, the pharmacological activities of many parts of mulberry were investigated such as antioxidative, anticancer, anti-inflammatory, antiobesity and neuroprotective activities [5,6,7,8,9,10,11]. In addition, mulberry is a good source of protein according to the Food and Agriculture Organization (FAO) and World Health Organization (WHO) recommendations [12]. Tryptophan is an essential amino acid that can be found in mulberry extracts. Besides being required for normal growth, tryptophan is a precursor for several bioactive compounds, including serotonin, melatonin, tryptamine, kynurenine and nicotinamide [13]. The pineal hormone, melatonin, predominantly participates in the regulation of circadian rhythms and sleep–wake cycles and possesses many pharmacological activities [14]. The consumption of tryptophan-enriched foods increased serum melatonin levels and the increasing melatonin improved sleep quality and elevated antioxidant capacities [15]. Therefore, these findings can categorize mulberries to be functional foods which benefit human health (Figure 1).

The caffeine-free tea made from mulberry leaves has gained interest in China, Japan and Korea. Because of the traditional medicine, mulberry leaf tea can promote health, relieve common cold, enhance eye, liver and kidney functions and decrease blood sugar and cholesterol [16,17]. In Thailand, mulberry is widely planted and the leaves that contain high levels of amino acids are used for sericulture and silk production. However, the old dark green leaves are only used for feeding silkworms. Previous reports of mulberry leaf tea processing found that total phenolic, flavonoid and melatonin contents were decreased [18,19]. Nevertheless, the change in tryptophan content in mulberry leaf tea has not been determined. Therefore, this study aims to investigate the content of tryptophan, melatonin, phenolics and flavonoids in fresh mulberry leaves and the processed leaf extracts from two different cultivars (cv.), including cv. Yai-Burirum (YB) and cv. Khunphai (KP). In addition, these extracts are also evaluated for their pharmacological activities such as antioxidant activities and acetylcholinesterase (AChE) inhibitory activity in vitro.

## 2. Results and Discussion

### 2.1. Total Phenolic and Flavonoid Contents of Mulberry Leaf Extracts

Mulberry leaves from two cultivars were prepared as fresh leaf (FL), green tea (GT) and black tea (BT) extracts. All extracts were evaluated to determine the content of phenolics and flavonoids, as shown in Table 1. Total phenolic content (TPC) in all samples ranged between 129.93 and 390.89 mg GAE/g extract. The heat processing of tea decreased the levels of TPC when compared to FL extracts in both cultivars. GT extracts also presented lower TPC than BT extracts. According to previous studies, the degradation in phenolic compounds was observed when the samples were exposed to high temperature [20,21]. Total flavonoid content (TFC) in all samples was found to range between 10.15 and 39.09 mg QE/g extract. The processing of tea increased the levels of TFC in GT and BT extracts in both cultivars. It was found that thermal exposure causes flavonoid degradation and transformation. The transformation generated the flavonoid-derived products and resulted in the increase in TFC [22]. However, the change in individual phenolics and flavonoids during tea processing was quantified by LS-MS/MS.

### 2.2. LC-MS/MS Analysis of Tryptophan, Melatonin, Phenolic and Flavonoid Contents

Tryptophan content in all samples was detected in the range 29.54 and 673.72 µg/g extract (Table 2). Interestingly, the processed mulberry leaves from both GT and BT extracts increased tryptophan content compared to FL extracts and BT extracts presented the highest amount among others in both cultivars. For cv. YB, the amount of tryptophan in the GT and BT extracts was about 4 and 23 times higher than the FL extracts, respectively. For cv. KP, tryptophan did not significantly change in the GT group, but it increased about 2 times in the BT group. In the tea manufacturing process, amino acids are produced by enzymatic activity through the proteolysis of proteins and peptides in tea leaves after plucking [23]. Moreover, proteins are also hydrolyzed by high temperature during tea processing and released free amino acids and volatile aromatic substances in tea are the results of amino acid transformation by heating process [24,25]. Therefore, the increase in an essential amino acid, tryptophan, would be the result of protein hydrolysis by high temperature during tea processing. Moreover, the enzymatic activity can be deactivated by steaming and results in different amounts of tryptophan in the GT and BT samples [23]. In addition, the intake of tryptophan-containing foods elevated the tryptophan level in the plasma within 90 minutes [26]. Tryptophan undergoes metabolic transformation to many bioactive metabolites including melatonin [15]. However, melatonin was not found in the extracts, except the fresh leaf extract of cv. YB. Obviously, melatonin would be reduced by the rolling and drying process. From a previous study, it also found that the exposure of high temperature during tea processing caused a loss of melatonin compared to fresh mulberry leaf extract [18].

Phenolic compounds that were analyzed and found in mulberry leaf extracts including gallic acid, caffeic acid, gentisic acid, protocatechuic acid and chlorogenic acid. Almost all phenolic acids were increased in the processed tea extracts except chlorogenic acid. Chlorogenic acid presented the highest amount in all samples. This compound presents many pharmacological activities such as antioxidant, anti-inflammatory, hepatoprotective and neuroprotective activities. Furthermore, it has been found that chlorogenic acid from the leaf extracts of *Morus* spp. reduced the levels of serum glucose, cholesterol and triglycerides in a dose-dependent manner [27,28]. In this study, chlorogenic acid slightly decreased in YB-GT extract, but sharply reduced in YB-BT extract. The reduction in chlorogenic acid in KP-GT and KP-BT was about 2 and 10 times. The change in chlorogenic acid affected total phenolic amounts in the extracts. For cv. YB, total phenolic compounds changed from 6612.60 ± 34.89 mg/g extract to 5916.91 ± 15.66 and 102.50 ± 1.48 mg/g extract in GT and BT, consecutively. For cv. KP, total phenolic compounds also decreased from 11,937.56 ± 151.67 mg/g extract to 5176.63 ± 52.89 and 1402.16 ± 10.29 mg/g extract in GT and BT, consecutively. Although the level of chlorogenic acid was lower in the processed tea, the content of this phenolic acid is high when compared to previous reports [18,29].

In this study, flavonoids including rutin and myricetin were quantified. Rutin was the only flavonoid that was detected in all extracts in the range 109.48–1009.75 mg/g extract. Attributed to the potent antioxidant properties of rutin, it was evaluated for biological activities such as anti-inflammation, antimicrobial and antitumor [30,31,32]. FL extracts from cv. YB and cv. KP showed higher levels than GT and BT extracts (1009.75 and 773.76 mg/g extract, respectively). Remarkably, about 90% and 50% of rutin decreased in the YB-GT and KP-GT groups compared to FL groups, while rutin in both BT groups was depleted by approximately 30% compared to FL extracts. Therefore, steaming significantly affected the degradation of rutin in tea processing. Thermal degradation of rutin generated new formed molecules which were identified such as protocatechuic acid, dimer of rutin, quercetin and quercetin-glucose [22]. The increase in protocatechuic acid in the GT and BT extracts would come from rutin thermal degradation. Unfortunately, myricetin was not detected in all extracts. Nevertheless, other flavonoids such as quercetin, isoquercetin, astragalin and kaempferol that have been discovered in mulberry leaves should be further investigated [33,34].

### 2.3. Antioxidant Capacities and In Vitro Acetylcholinesterase (AChE) Inhibition of Mulberry Leaf Extracts

Antioxidative capacities of FL, GT and BT extracts were evaluated by DPPH, ABTS and FRAP assays. The radical scavenging abilities of processed tea samples in DPPH and ABTS assays were different (Table 1). The scavenging activities of YB-GT and YB-BT extracts were slightly decreased compared to YB-FL; meanwhile, KP-GT and KP-BT scavenging effects slightly increased. For the reducing power evaluation by FRAP assay, it found that the extracts had FRAP value between 111.62–239.26 mmol/100 g extract. Tea processing elevated the FRAP value of GT and BT in both cultivars. Therefore, the change in phenolic and flavonoid compounds from the heating process resulted in the antioxidant capacities of the GT and BT extracts.

Currently, therapeutic drugs approved for Alzheimer’s disease are focused on two mechanisms, including the agonism of the cholinergic system and antagonism of the N-methyl-D-aspartate receptor (NMDA-receptor) [35]. In this study, the inhibitory effect of the extracts on AChE activity was evaluated. The results found that all extracts presented the inhibitory activity with IC_50_ in the range 146.53–165.24 µg/mL. Both GT and BT extracts slightly increased AChE inhibitory effect of GT and BT extracts. Besides the antioxidant activity, phenolics and flavonoids also contribute to the inhibitory effect on AChE [36,37,38]. Therefore, the processing tea influenced the AChE inhition due to the change in phenolic and flavonoid contents.

## 3. Materials and Methods

### 3.1. Sample Preparation and Extraction

Tips and young leaves (1st–6th leaves from the top of each branch) of two mulberry cultivars were harvested in June 2018 from The Queen Sirikit Department of Sericulture, Khon Kaen Province, Thailand. The leaves were washed, dried and ground. The powdered samples (500 g) were extracted by ultra-sonication with methanol (1000 mL) for 20 min. Then, the extracts were filtered, evaporated with a rotary evaporator and freeze dried [39]. The extraction was carried out in triplicate.

**Figure 2:**
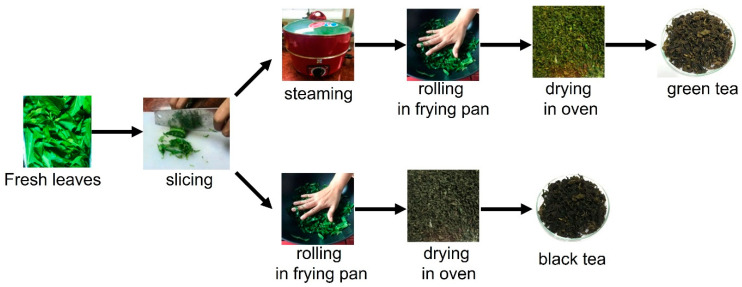
Scheme of tea processing in this study.

The preparation of green and black tea from mulberry leaves was modified from the study of Pothinuch and Tongchitpakdee [19]. After washing and drying, the leaves were sliced into the size 0.5 × 0.5 cm^2^. The leaves were divided into two groups. For GT, the leaves were steamed for 1 min, rolled in the pan for 30 min and dried in the oven at 80 °C for 1 hour. Without steaming, the leaves were kneaded in the pan and dried in the oven at 80 °C for 1 h to obtain BT (Figure 2). Then, the extraction of tea samples was carried out the same as the fresh leaf extraction. The yields of FL, GT and BT methanolic extracts are in the range 2.13–5.30%, as shown in Table 3.

### 3.2. Tryptophan, Melatonin and Phenolic Determination by LC-MS

Melatonin and tryptophan were determined by liquid chromatography–mass spectrometry (LCMS 8030 Triple Quadrupole Mass Spectrometer, Shimadzu Corp, Kyoto, Japan). The chromatographic separations were performed on an Insertsil ODS-3 C18 (GL Sciences Inc., Japan (150 × 2.1 mm i.d., 3 µm) using the mixture of 0.45% formic acid in water:acetonitrile (1:1, *v*/*v*) at a flow rate of 0.2 mL/min at 35 °C. The sample injection volume was 2 µL. The electrospray source had the following settings: drying gas (N_2_) flow of 15 L/min at 350 °C, nebulizing gas flow at 3 L/min and positive capillary interface voltage of 4.5 kV. MS data were acquired in the positive mode. Melatonin and tryptophan were identified by multiple reaction monitoring (MRM). The transitions of precursor to product of molecular ion were determined from *m*/*z* 233 to *m*/*z* 174 for melatonin and *m*/*z* 205 to *m*/*z* 188 for tryptophan. A dwell time was set at 100 ms for each transition. The method was modified from Kim et al. [40]. 

Quantification of phenolic and flavonoid compounds was modified from a previous method [41]. The mobile phase for phenolic (gallic acid, caffeic acid, gentisic, protocatechuic acid and chlorogenic acid) and flavonoid (rutin and myricetin) determination consisted of 1% acetic acid in water and acetonitrile in ratio 1:1 with flow rate 0.2 mL/min with column C-18 (150 × 2.1 mm, 3 µm) at 40 °C and injection volume as 2 µL. The MS detection was operated on a triple quadrupole mass spectrometer with negative electrospray ionization (ESI) mode (ion source temperature of 400 °C) with MRM and monitored mass transitions of gallic acid (*m/z* 169 to 125), caffeic acid (*m*/*z* 179 to 135), gentistic acid (*m*/*z* 153 to 108), protocatechuic (*m*/*z* 153 to 109), chlorogenic acid (*m*/*z* 353 to 191), rutin (*m*/*z* 609 to 300) and myricetin (*m*/*z* 317 to 151).

### 3.3. Determination of Total Phenolic Content (TPC)

The total phenolic content of mulberry fruit extract was evaluated using Folin–Ciocalteu colorimetric method. The samples (20 µL) were mixed with 10% Folin–Ciocalteu reagent (100 µL) in a 96-well plate and incubated for 5 min. Then, a 7% sodium carbonate (80 µL) solution was added into the mixture and incubated at room temperature for 30 min. The mixture was measured at 700 nm by microplate reader. The total phenolic content was calculated as gallic acid equivalent units (mg GAE/g extract) [42].

### 3.4. Determination of Total Flavonoid Content (TFC)

The samples (100 µL) were mixed with 2% aluminum chloride solution (100 µL) in a 96-well plate. Then, the mixture was measured at 415 nm by microplate reader. The total flavonoid content was calculated as quercetin equivalent units (mg QE/g extract) [43].

### 3.5. Antioxidant Activities

#### 3.5.1. 1,1-Diphenyl-2-picrylhydrazyl (DPPH) Radical Scavenging Assay

The 1,1-diphenyl-2-picrylhydrazyl (DPPH) was dissolved in ethanol at a concentration of 200 µM. The 200 μM DPPH was mixed with a different concentration of samples (100 μL each) on a 96-well plate and incubated in dark conditions at room temperature for 30 min. The absorbance of the mixture was measured by a microplate reader at 517 nm. Trolox was used as the positive control. The IC_50_ was calculated by plotting between the percentage of inhibition and concentration of samples [44].

#### 3.5.2. 2,2-Azinobis (3-ethylbenzothiazoline-6-sulfonic acid) (ABTS) Radical Scavenging Assay

The ABTS radical cation was prepared by potassium persulfate followed by 7 mM ABTS which was mixed with 2.45 nM potassium persulfate in purified water. The ABTS reagent was kept at room temperature in the dark place for 12 h before use. The sample (100 μL) was mixed ABTS (100 μL) in a 96-well plate and incubated at room temperature for 10 min. The absorbance of the mixture was measured by a microplate reader at 700 nm. Trolox was used as the positive control. The IC_50_ was calculated by plotting between the percentage of inhibition and concentration of samples [45].

#### 3.5.3. Ferric Reducing Antioxidant Power (FRAP) Assay

FRAP reagent prepared by the mixture of 300 mM acetate buffer (pH 3.6), 20 mM FeCl_3_ solution and 10 mM TPTZ in 40 mM HCl in ratio of 10:1:1. The samples (20 μL) were mixed with FRAP reagent (80 μL) in a 96-well plate. The mixture was incubated at 37 °C for 4 min. The absorbance of the mixture was measured by a microplate reader at 595 nm. Trolox was used as the positive control. The FRAP values were shown in (mmole)/100 g extract [46].

### 3.6. In Vitro Inhibition Study on Acetylcholinesterase (AChE) Enzyme

The measurement of AChE inhibitory effect was modified from the spectrophotometric assays described by Ellman [47] and Petrachaianan [48]. Shortly, 125 µL of 3 mM 5,5′-dithiobis-(2-nitrobenzoic acid) (DTNB), 25 µL of 1.5 mM acetylthiocholine iodide (ATCI) and 25 µL of sample were dissolved in 50 µL of 50 mM TRIS-HCl buffer pH 8.0 and added to the wells. Then, 25 µL of 0.28 U/mL AChE from Electrophorus electricus was added. The absorbance was measured at 405 nm by a microplate reader (Bio-Tek Instrument, Winooski, VT, USA). Three independent experiments were performed and each experiment was run in triplicate.

### 3.7. Statistical Analysis

The data were shown in mean ± standard deviation (SD). The data were analyzed using one-way analysis variance (ANOVA) with SPSS 19.0 software for Windows. Tukey HSD test was used to determine the significant differences between samples (*p* < 0.05).

## 4. Conclusions

Considering that mulberry is a good source of bioactive compounds, these compounds play an important role in promoting human health. Apart from silkworm feeding, the use of mulberry leaves for processed tea would add to the value of the products. The amount of tryptophan that was found in the mulberry leaf tea suggested that it could be a promising source of tryptophan. Although the heating process caused the decrease in phenolic acids, especially chlorogenic acid, the total phenolic compounds in the tea extracts were high. Moreover, the tea extracts also had antioxidative and anti-AChE activities. Therefore, processed tea from mulberry leaves could serve as a new alternative functional food for health-concerned consumers. 

## Figures and Tables

**Table 1:** Antioxidant activities and AChE inhibitory effect of mulberry leaf extracts.

Samples	TPC (mg GAE/g Extract)	TFC (mg QE/g Extract)	Antioxidant Capacities			AChE Inhibition IC_50_ (µg/mL)
			DPPH IC_50_ (µg/mL)	ABTS IC_50_ (µg/mL)	FRAP (mmole/100 g Extract)	
YB-FL	390.89 ± 3.90 ^a^	10.15 ± 0.21 ^c^	96.17 ± 2.28 ^b^	86.05 ± 1.40 ^b^	163.99 ± 9.26 ^b^	165.24 ± 5.11 ^c^
YB-GT	194.69 ± 2.81 ^c^	29.42 ± 1.06 ^b^	253.70 ± 1.71 ^d^	145.22 ± 6.20 ^c^	143.15 ± 0.44 ^c^	160.66 ± 6.51 ^c^
YB-BT	261.10 ± 1.69 ^b^	39.09 ± 0.48 ^a^	117.69 ± 0.90 ^c^	90.40 ± 2.18 ^b^	187.54 ± 0.94 ^a^	146.53 ± 2.66 ^b^
KP-FL	290.94 ± 4.56 ^a^	17.59 ± 0.23 ^b^	128.97 ± 1.17 ^d^	88.71 ± 1.47 ^d^	111.62 ± 1.96 ^c^	163.07 ± 2.30 ^c^
KP-GT	129.93 ± 2.37 ^c^	30.76 ± 0.05 ^a^	121.55 ± 1.23 ^c^	76.89 ± 1.32 ^c^	173.52 ± 1.29 ^b^	156.01 ± 3.19 ^b^
KP-BT	252.15 ± 4.72 ^b^	30.24 ± 0.17 ^a^	117.75 ± 1.42 ^b^	67.35 ± 1.15 ^b^	239.26 ± 1.78 ^a^	159.51 ± 4.24 ^b,c^
Trolox	-	-	3.40 ± 0.01 ^a^	4.49 ± 0.04 ^a^	40.89 ± 0.51 ^d^	-
Galantamine	-	-	-	-	-	1.06 ± 0.08 ^a^

Letters indicate the significant difference in data between rows in the same columns at *p* < 0.05, using one-way ANOVA with Tukey HSD.

**Table 2:** Quantification of phenolic and flavonoid compounds from mulberry leaf extracts by LC-MS analysis.

Samples	Tryptophan (µg/g Extract)	Melatonin (µg/g Extract)	Phenolics (mg/g Extract)					Flavonoids (mg/g Extract)	
			Gallic Acid	Caffeic Acid	Gentisic Acid	Protocatechuic Acid	Chlorogenic Acid	Rutin	Myricetin
YB-FL	29.54 ± 4.03 ^c^	9.77 ± 0.32	1.84 ± 0.08 ^c^	7.67 ± 0.15 ^b^	17.58 ± 0.27 ^c^	4.91 ± 0.45 ^c^	6580.60 ± 35.28 ^a^	1009.75 ± 11.29 ^a^	ND
YB-GT	113.03 ± 2.6 ^b^	ND	5.95 ± 0.32 ^a^	4.78 ± 0.88 ^c^	50.84 ± 1.84 ^b^	22.51 ± 1.06 ^a^	5832.83 ± 15.82 ^b^	109.48 ± 1.55 ^c^	ND
YB-BT	673.72 ± 4.43 ^a^	ND	3.23 ± 0.47 ^b^	11.91 ± 1.00 ^a^	54.00 ± 0.79 ^a^	13.73 ± 0.38 ^b^	19.63 ± 0.92 ^c^	751.33 ± 13.80 ^b^	ND
KP-FL	209.40 ± 11.87 ^b^	ND	2.49 ± 0.27 ^a^	5.80 ± 0.14 ^c^	16.13 ± 0.54 ^c^	6.18 ± 0.13 ^c^	11907.10 ± 151.80 ^a^	773.76 ± 26.20 ^a^	ND
KP-GT	210.86 ± 1.38 ^b^	ND	2.54 ± 0.13 ^a^	7.77 ± 0.74 ^b^	36.49 ± 2.35 ^b^	7.75 ± 0.14 ^b^	5122.08 ± 50.75 ^b^	372.66 ± 6.90 ^c^	ND
KP-BT	481.49 ± 10.11 ^a^	ND	2.65 ± 0.24 ^a^	11.17 ± 0.80 ^a^	46.93 ± 1.31 ^a^	10.73 ± 0.53 ^a^	1330.69 ± 11.36 ^c^	543.14 ± 4.04 ^b^	ND

Letters indicate the significant difference in the data between rows in the same columns at *p* < 0.05, using one-way ANOVA with Tukey HSD.ND = not detected.

**Table 3:** Weights and yields of mulberry leaf extracts.

Samples	Fresh Weights (g)	Extracts (g)	Yields (%)
YB-FL	500.00	23.12	4.63
YB-GT	100.00	3.40	3.40
YB-BT	100.00	2.13	2.13
KP-FL	500.00	26.43	5.30
KP-GT	100.00	3.07	3.07
KP-BT	100.00	2.93	2.93

## Data Availability

All data that support the findings of this study are available within the article.
